# Packaging Reliability Effect of ENIG and ENEPIG Surface Finishes in Board Level Thermal Test under Long-Term Aging and Cycling

**DOI:** 10.3390/ma10050451

**Published:** 2017-04-26

**Authors:** Chaobo Shen, Zhou Hai, Cong Zhao, Jiawei Zhang, John L. Evans, Michael J. Bozack, Jeffrey C. Suhling

**Affiliations:** Center for Advanced Vehicle and Extreme Environment Electronics (CAVE3), Auburn University, Auburn, AL 36849, USA; jhai@nvidia.com (Z.H.); czz0025@auburn.edu (C.Z.); zhangjiawei19831010@gmail.com (J.Z.); evansjl@auburn.edu (J.L.E.); bozacmj@auburn.edu (M.J.B.); jsuhling@auburn.edu (J.C.S.)

**Keywords:** thermal cycling, surface finishes, SAC, ENIG, ENIPIG, intermetallic, Ag_3_Sn, crack propagation

## Abstract

This study illustrates test results and comparative literature data on the influence of isothermal aging and thermal cycling associated with Sn-1.0Ag-0.5Cu (SAC105) and Sn-3.0Ag-0.5Cu (SAC305) ball grid array (BGA) solder joints finished with ENIG and ENEPIG on the board side and ENIG on the package side compared with ImAg plating on both sides. The resulting degradation data suggests that the main concern for 0.4 mm pitch 10 mm package size BGA is package side surface finish, not board side. That is, ENIG performs better than immersion Ag for applications involving long-term isothermal aging. SAC305, with a higher relative fraction of Ag_3_Sn IMC within the solder, performs better than SAC105. SEM and polarized light microscope analysis show cracks propagated from the corners to the center or even to solder bulk, which eventually causes fatigue failure. Three factors are discussed: IMC, grain structure, and Ag_3_Sn particle. The continuous growth of Cu-Sn intermetallic compounds (IMC) and grains increase the risk of failure, while Ag_3_Sn particles seem helpful in blocking the crack propagation.

## 1. Introduction

Lead-free solder is commonly used in electronic packaging industries to eradicate the harmful effects of Pb on human health and the ecosystem [1]. The performance and quality of the solder are crucial to the integrity of a solder joint, which in turn is vital to the overall functioning of the assembly [2]. Among several candidate alloys, SAC series alloys have emerged as the most widely accepted because of its relatively low melting temperature, its superior mechanical properties, and its relatively good wettability [3]. Research has been driven by the varying reliability of performance of near-eutectic SAC alloys under different stimuli—such as impact, vibration and thermo-mechanical loading [4]. For example, Shnawah et al. [5] evaluated the SAC105 and SAC305 solder ball joints for BGA interconnections under drop impact tests and found SAC105 solder balls showed better performance than SAC305.

Many studies pay attention to the mechanical property change and microstructural evolution of solder joints subjected to accelerated mechanical test conditions such as drop, vibration, and short-term aging tests [6,7,8]. However, long term thermal cycling or thermo-mechanical fatigue testing of solder joints is also very important for the reliability of solder joints. Because of the large difference in the coefficient of thermal expansion (CTE) of the different constituents in the packaged assembly, stresses and strains vary with temperature leading to cyclic strain or inelastic energy damage and fatigue failure of the solder joints [9]. 

For SAC alloys, there are studies that indicate that the creep and fatigue behavior can be strongly influenced by microstructure, surface finish, shear and thermal cycle profile. The choice of PCB surface finish is a very important decision. It impacts assembly capability, PCB shelf life, solder joint reliability, cost and on-time delivery performance. Nowadays, Electroless plating is a more suitable finish for electronic parts as they continue down the path of smaller and lighter. Electroless Nickel/Immersion Gold (ENIG) is commonly used for substrates that require soldering and mechanical contacting. Electroless Nickel/electroless palladium/ Immersion Gold (ENEPIG) has excellent solderability for Sn-Ag-Cu based solders and forms high reliability wire bonds [10]. The purpose of this work is to discover the long term isothermal performance of Sn98.5Ag1.0Cu0.5 (SAC105) and Sn96.5Ag3.0Cu0.5 (SAC305) solder interconnects fabricated on ENIG and ENEPIG finished printed wiring boards. The Ni layer inside of ENIG and ENEPIG has a toughening effect on the intermetallic thereby inhibiting crack formation and growth [11,12]. The failure data obtained are also compared to previous study on immersion silver (ImAg) surface finish subject to the identical testing conditions. Details of the cycles to failure test results and degradation charts to above 4000 cycles and the effect of intermetallic compound growth on solder joints failures are provided in the following sections.

## 2. Experiment

**Figure 1:**
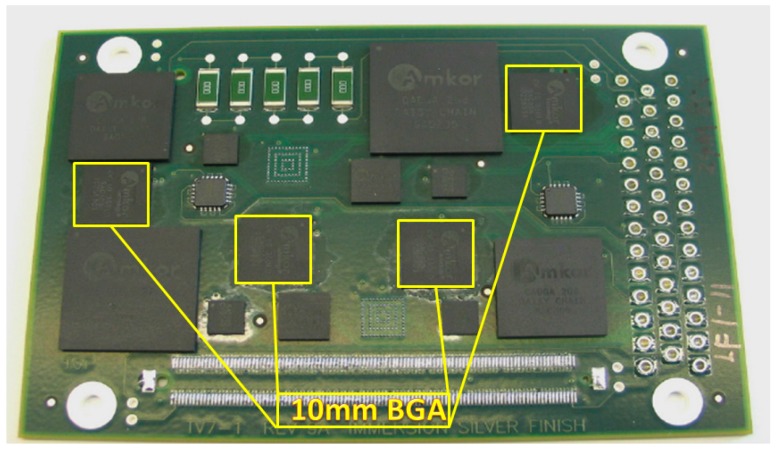
Assembled Test Vehicle.

The assembled test vehicle is shown in Figure 1. The assembly test boards were four-layer FR-4 glass epoxy PCBs with dimensions 100.076 mm × 67.056 mm having a glass transition temperature (Tg) of 170 °C. Each test board was populated with 5 mm × 5 mm, 10 mm × 10 mm, 15 mm × 15 mm, and 19 mm × 19 mm BGA, 7 mm × 7 mm CSP, 5 mm × 5 mm MLF, and a series of 2512 resistors.

The discussion mainly focuses on the 10 × 10 mm CVBGA component package. The detail information of 10 mm BGA is listed as Table 1. For these test specimens, the solder paste matched the solder composition of solder spheres on the BGA packages.

**Figure 2:**
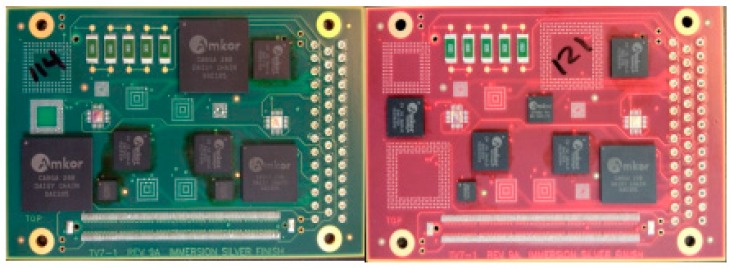
ENIG (**Left**) and ENEPIG (**Right**).

To help identify the board with different finishes, we apply two different colors for the PCB board. The green one is ENIG, the red one is ENEPIG (Figure 2).

**Figure 3:**
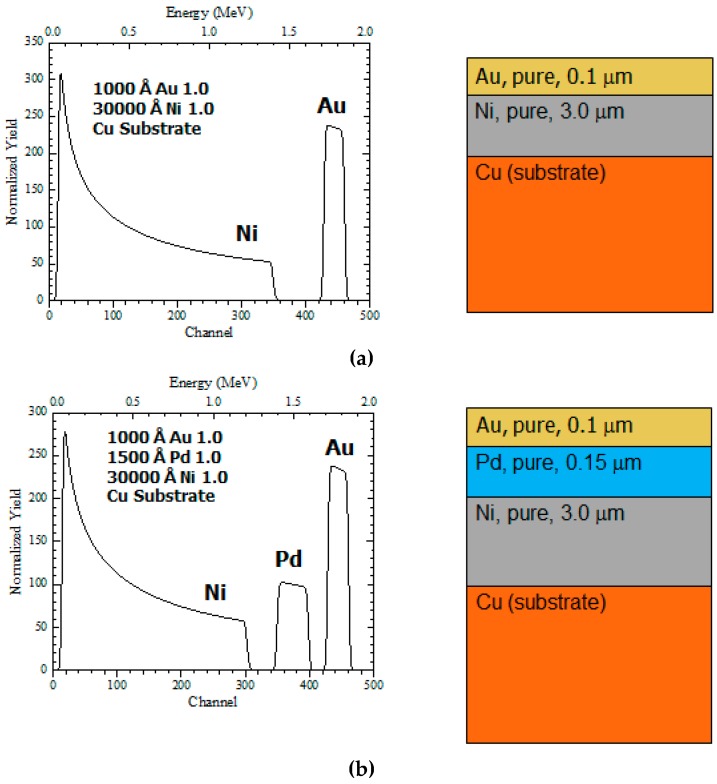
RBS Analysis for ENIG (**a**) and ENEPIG (**b**).

Rutherford Backscattering Spectroscopy (RBS) is conducted for ENIG and ENEPIG to measure the thickness of metallic layers, see Figure 3. The ENIG finish consists of two metallic layers: a thin gold coating over the thicker nickel coating deposited via electroless process directly onto the PCB copper pad. The additional 0.15 um of Pd layer from ENEPIG improves the wettability and acts as a diffusion barrier.

Table 2 provides details regarding the test plan and sample size (number of components) used in this study. Three surface finishes—ImAg, ENIG, and ENEPIG—were employed. The test vehicles were grouped in time periods of 0, 6, and 12 months and subjected to 125 °C aging temperature. 

**Figure 4:**
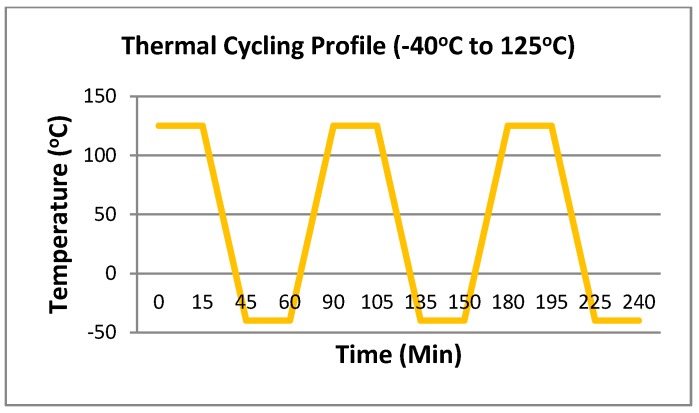
Thermal Cycling Profile.

The boards were placed vertically in the accelerated thermal cycling furnace and wired to a switch scanning system with a high accuracy digital multi-meter which continuously monitored the resistance change of each component. Based on IPC-9701, the practical definition of solder joint failure is an interruption of electrical continuity >1000 Ohms. In this study, failure was defined to be the point when the daisy chain resistance was >300 Ohms for five repeated resistance measurements. The thermal test profile was set to run with a 15 min dwell time and 30 min ramp time with the temperature ranging from −40 °C to 125 °C. See Figure 4.

All samples for microscopic examination were cross-sectioned and polished with SiC polishing paper (240, 320, 400, 600, 800, 1200), followed by 0.1 μm and 0.05 μm micron diamond suspension fine polishing. SEM back-scattered electron images and polarized light images were captured from specimens to observe the IMCs and microstructure of the solder joints.

## 3. Result and Discussion

### 3.1. Data Analysis

The failure life of electronic packages is characterized with a Weibull distribution. The characteristic life (η) is the point (i.e., number of cycles) at which 63.21% of the population is expected to fail. The slope (β) of the Weibull distribution distinguishes different classes of failure modes. These Weibull results are tabulated in Table 3 [13]. After aging at 125 °C for 6 months, the characteristic lifetime for 10 mm SAC305 solder on ENIG, ENEPIG, and ImAg drops from 3974, 4661 and 3329 cycles to 2894, 2635 and 1740 cycles, respectively. And further, it reduced to 2671, 2536, and 1551 cycles subjected to 125 °C aging for 12 months.

**Figure 5:**
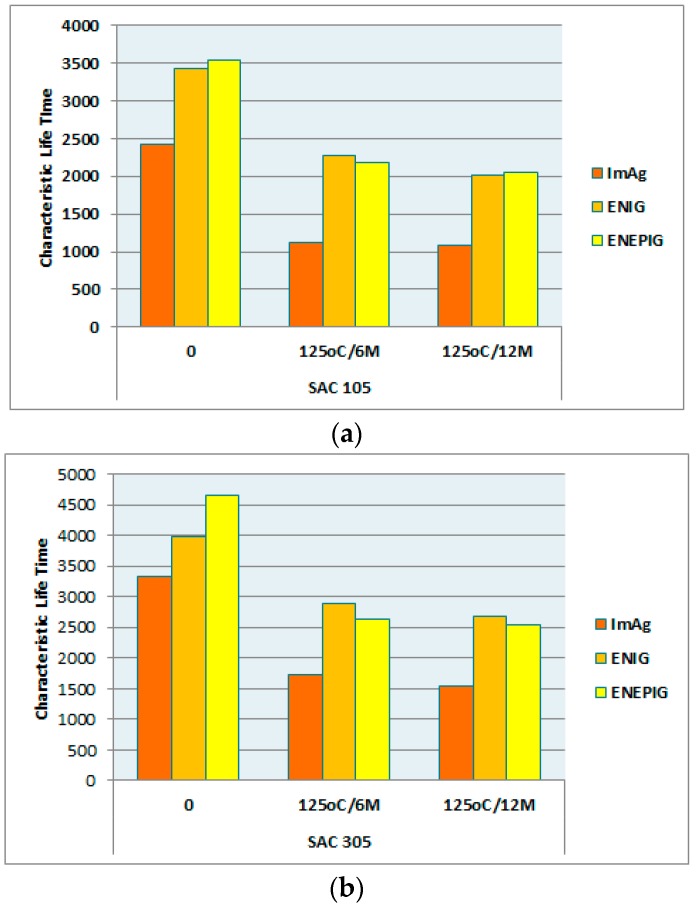
Characteristic Life Comparison (η) for 10 mm BGA finished with ENIG, ENEPIG, and ImAg subject to 125 °C/12 months aging. (**a**) SAC105 (**b**) SAC305.

Based on the Weibull characteristic life (η), Figure 5 gives a comparison of the reliability performance among surface finishes for 10 mm packages (0.4 mm pitch) with 125 °C/12 months aging. In most cases, the rank of the characteristic lifetimes for both SAC105 and SAC305 with different surface finishes after aging can be ordered as: ENIG ≈ ENEPIG > ImAg.

**Figure 6:**
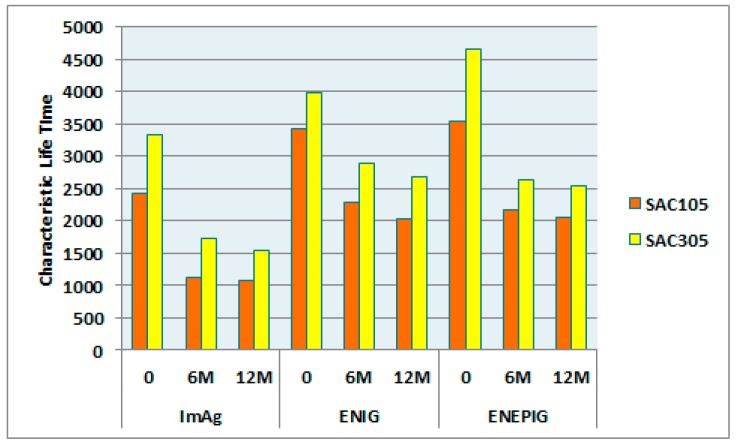
Characteristic Lifetime Comparison (η) for 10 mm BGA soldered with SAC105 and SAC305 subject to 125 °C/12 months aging.

Figure 6 provides a comparison of the reliability performance between SAC105 and SAC305 for the 10-mm package (0.4 mm pitch) subject to 125 °C aging. We conclude that SAC305 solder performs better than SAC105 in all cases, and illustrates the risk in using SAC105 solder balls in applications where thermal fatigue failure is a concern.

**Figure 7:**
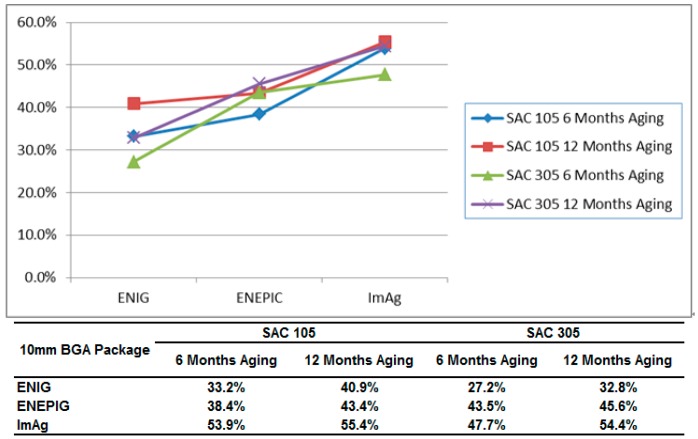
Degradation Rate Comparison for 10 mm BGA soldered with SAC105 and SAC305 subject to 125 °C/12 months aging.

In Figure 7, the degradation rate comparison indicates that ENEPIG drops faster than ENIG after 6 months and 12 months of aging. For example, in 6 months aging, sac 105 group, ENIG’s degradation rate is 33.2%, and ENEPIG’s degradation rate is 38.4%.

### 3.2. Failure Analysis

Previous study from Zhou et al. found the thickness of the IMC layer at the interface between the solder and substrate is very important in determining the reliability of the whole package. Formation of the intermetallic compound (IMC) layers at the interface is an indication of good bonding between solder and the metal pad. However, an excessively thick IMC layer is sensitive to stress and sometimes provides initiation places and paths for the propagation of cracks [14]. Thus, it is essential to study the formation and growth of the IMC layer, as the growth of the IMC layer could degrade the reliability of the solder joint.

#### 3.2.1. Intermetallic (IMC)

Prior investigations [15,16] in many laboratories have shown that the continuous growth of interfacial intermetallic during isothermal aging strongly influences fatigue failure of solder joints. As the brittle intermetallic layer consumes a larger fraction of the solder ball, cracks propagate along them, causing solder joint cracking and eventual failure under the high strain due to CTE mismatches.

**Figure 8:**
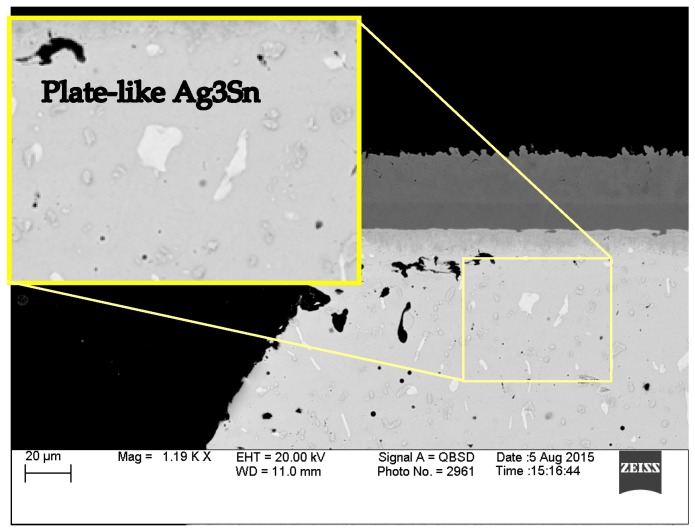
Plate-like Ag_3_Sn.

In Figure 8, large, plate-like Ag_3_Sn phases are found regularly in SAC305 solder joints subject to isothermal aging alone, which is due to its higher Ag content. Zhang et al. observed that coarsened Ag_3_Sn IMC reduces/redirects the crack growth which results in enhanced joint structural strength [17,18]. This helps to explain the superior thermal fatigue performance of SAC305 (higher Ag content material) than SAC105.

For the SAC solder with ImAg, a first layer Cu_6_Sn_5_ (η-phase) intermetallic is formed at the board/solder joint interface. Then, a second layer of Cu_3_Sn (ε-phase) is formed at the IMC layer/board interface during aging which reduces the mechanical behavior of the solder joint. After the Cu atoms arrive at the interface of Cu_3_Sn/Cu_6_Sn_5_ by diffusion through the grain boundaries of the Cu_3_Sn layer, the following interfacial reaction happens:

Cu_6_Sn_5_ + Cu → Cu_3_Sn

By this reaction, Cu_6_Sn_5_ is converted to Cu_3_Sn at the interface [19].

**Figure 9:**
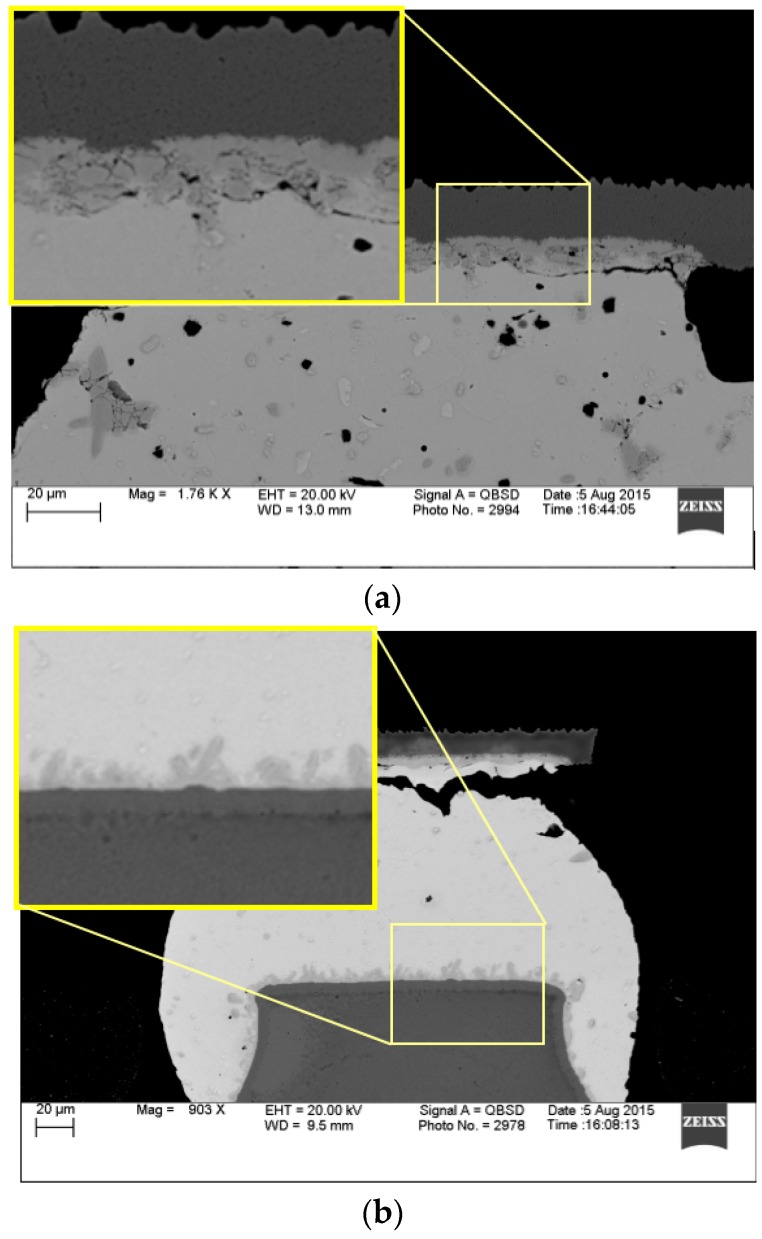
IMC layer at package side (**a**) and board side (**b**).

For ENIG/ENEPIG finishes, as Figure 9 shows, in the interfacial reaction between the Ni layer and SAC 105 /SAC 305, ternary IMCs consisting of Cu, Ni, and Sn are observed. The IMC layer is of Cu–Sn type with a small proportion of nickel: (Ni,Cu)_6_Sn_5_ and (Ni,Cu)_3_Sn_4_ [20]. The nickel layer in ENIG/ENEPIG systems acts as a diffusion barrier which inhibits Cu dissolution into the solder to ensure better reliability. Phosphorus is also present, due to the process deposition of the nickel layer. In our test, all components are finished with ENIG on the package side.

**Figure 10:**
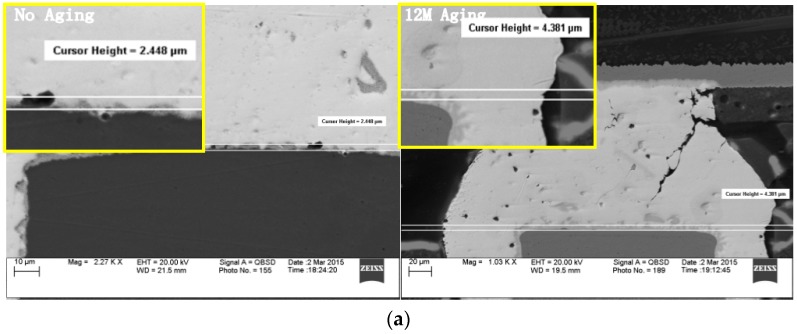

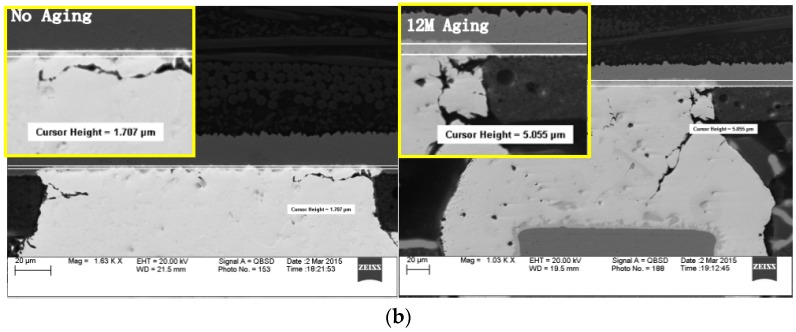
IMC thickness growth with the effect of aging for ENIG (**a**) board side; (**b**) package side.

Moreover, recent investigations [21] had reported that the growth of Cu–Sn IMC layer had a degraded effect on the solder joint reliability, with increasing thickness of the Cu–Sn IMC layer, the thermal fatigue life of solder joints will decrease. Figures in blow show the IMC thickness growth after aging. (Figure 10)

#### 3.2.2. Crack Analysis

**Figure 11:**
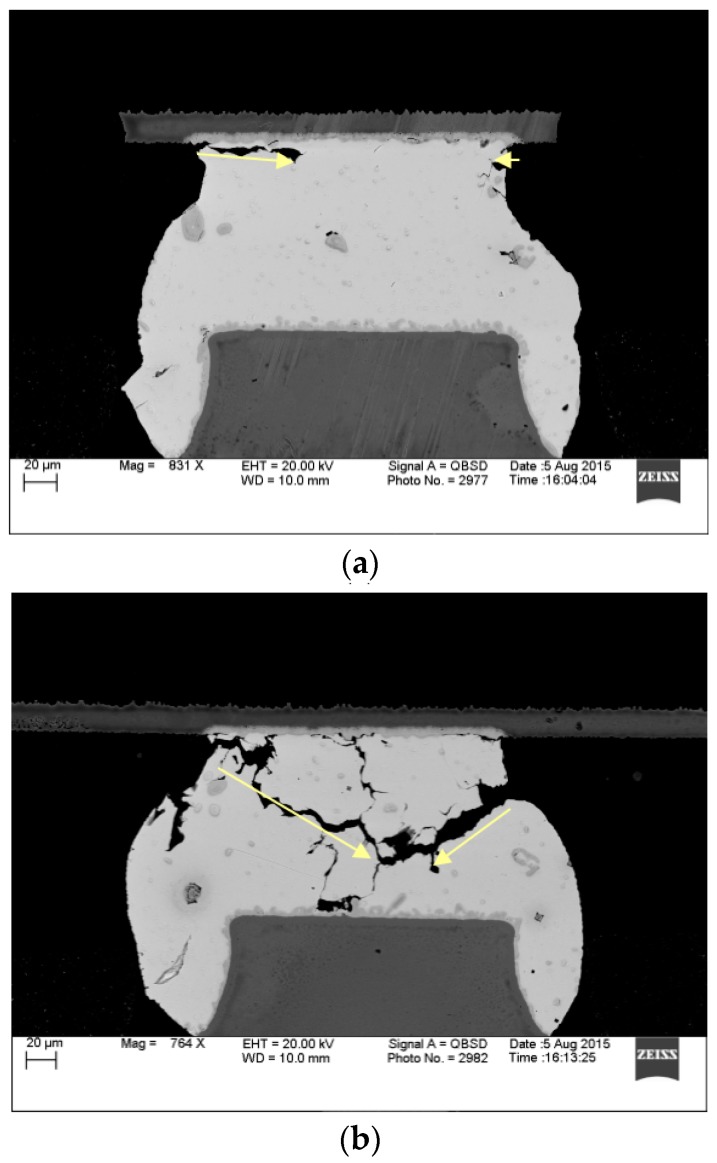

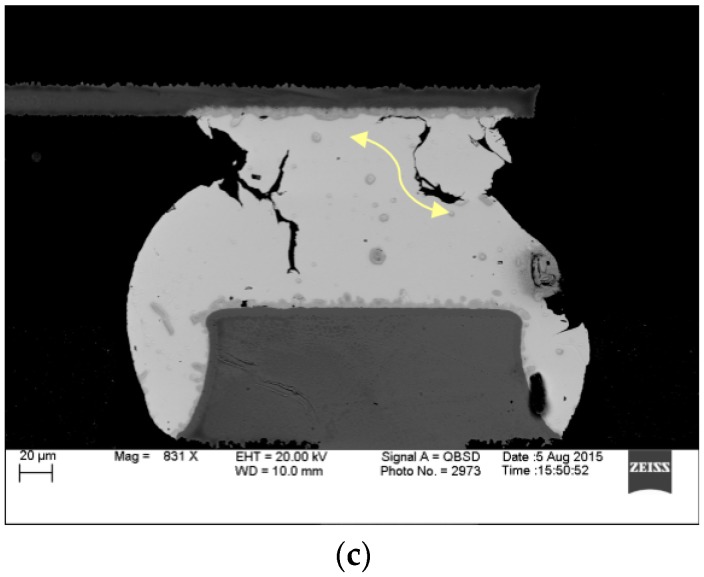
SEM images of 10 mm package SAC105 solder interconnections after 12 months of aging at 125 °C: (**a**) Cracks at the package side going along with the IMC boundary, ENEPIG finished; (**b**) Cracks go into the solder ball, ENIG finished; (**c**) A crack generates from the inside of solder ball, ENIG finished.

The shear stress created from the CTE mismatches at the both package and board-side solder/finish interface cause plastic deformation, which generates cracks which propagate along those areas of the solder joint [22]. Figure 11 shows three typical failure modes for the 10-mm package size, 0.4 mm pitch BGA after thermal aging and cycling for SAC105 on ENEPIG and ENIG surface finishes, respectively. It looks like the microstructure deformation of finer pitch solder interconnections (0.4 mm) is much more severe. The cracks are more likely initiated at the package side corner of the interconnection and then proceed along the IMC boundary, see Figure 11a. Figure 11b shows a crack path with an angle downwards to the solder bulk after thermal aging and cycling. Occasionally, cracks may generate from the inside of solder ball (Figure 11c).

**Figure 12:**
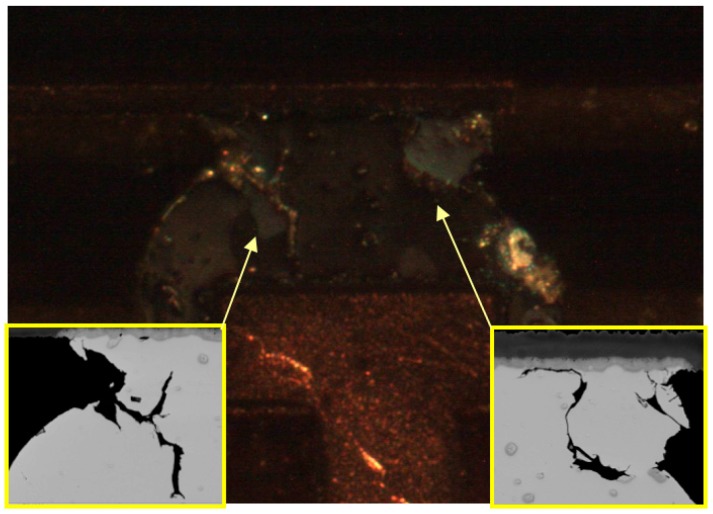
Cross-polarized image of Figure 11c.

As Figure 12 shows, the reason that cracks generated from the inside of solder ball appears to be the grain structure recrystallization. It is clear that the fatigue failure comes up and propagated at grain boundaries.

Finer pitch packages typically have less structural stability than larger pitch packages. Figure 12 shows the cross-polarized image of 10 mm SAC105 solder balls, the crack which might be effect by grain boundaries. This is due to the formation of a continuous network of high-angle (grain) boundaries by local recrystallization increasing grain structure movement along the interfacial region, enhancing cracks nucleation and propagation through the recrystallized solder interconnections [23,24].

**Figure 13:**
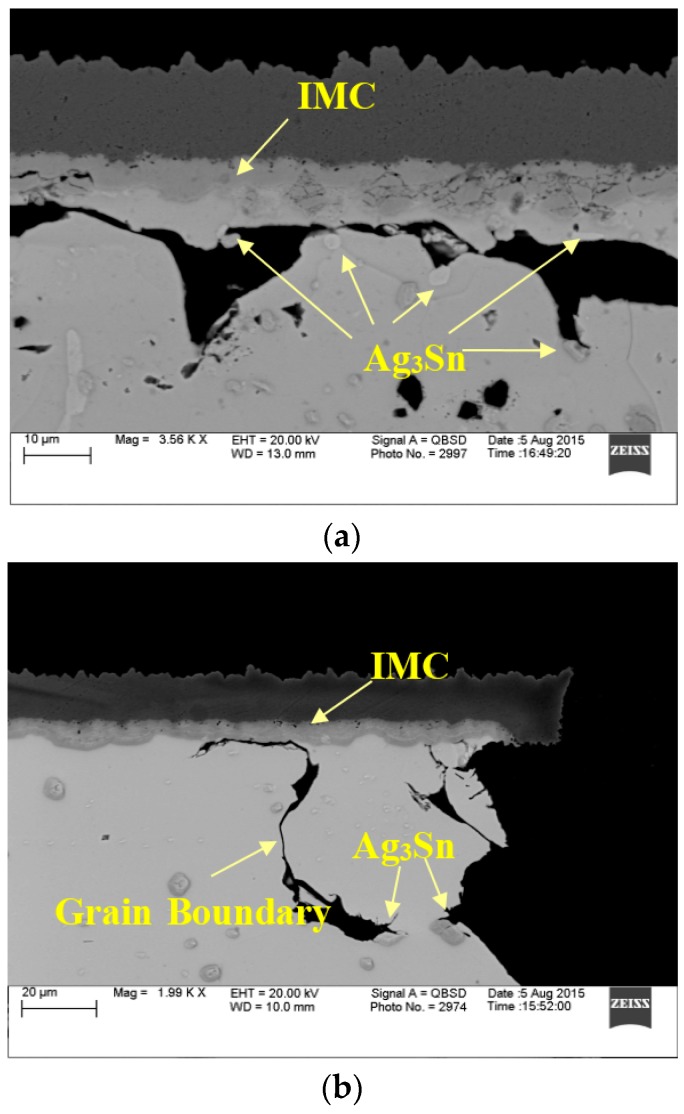
SEM images of 10 mm package SAC105 solder interconnections after 6 months of aging at 125 °C: (**a**) Crack goes along with the IMC boundary and blocked by Ag_3_Sn particles, ENEPIG finished; (**b**) Crack propagation is subject to the IMC boundary, grain boundary, and Ag_3_Sn particles. ENIG finished.

Figure 13 shows another two factors: (1) package side IMC boundaries; (2) larger IMC precipitates in the solder bulk as Ag_3_Sn.

**Figure 14:**
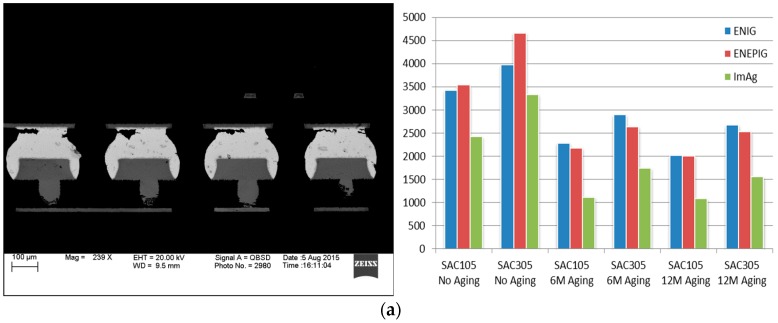

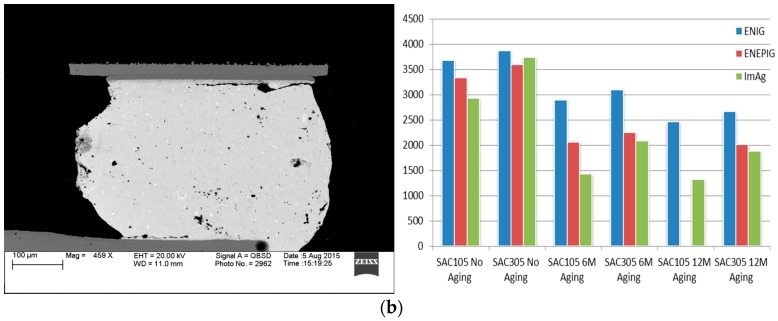
Failure performance for different board finishes on (**a**) 10 mm BGA and (**b**) 15 mm BGA. (Y axis: cycle time).

In my previous work about the 15 mm (0.8 mm pitch) package, the crack is always happened at both sides. The Weibull analysis solution gives the conclusion that board side surface finish ENIG performance better than ENEPIG [25]. However, after the above failure analysis, we find that, for the 10 mm (0.4 mm pitch) package, cracks normally happened at the package side, not the board side. The data plots also show the gap between ENIG and ENEPIG is very tiny for 6 months and 12 months aging (Figure 14). That means package side surface finish is the determining factor. Actually, the conclusion we can get is package side surface finish ENIG has better performance than ImAg. Pitch size may be a good concern for choosing surface finish.

## 4. Conclusions

The Weibull characteristic lifetime is dramatically reduced during isothermal aging at 125 °C for SAC105/305 on a variety of surface finishes. In particular, SAC105 undergoes a considerable lifetime reduction during aging and illustrates the risk in using SAC105 solder balls in applications where thermal fatigue failure is a concern. Generally, the rank of the characteristic lifetimes for both SAC105/305 with different board finishes follows the order as: ENIG ≈ ENEPIG > ImAg. The gap between ENIG and ENEPIG is very tiny. In the further crack analysis, we find most of cracks happened at the package side for the 10 mm BGA. The main concern of failure is package side surface finish. Both ENIG and ENEPIG board plating groups are applied with the same package side surface finish-ENIG, and ImAg is plating on both sides. Thus, it comes to the conclusion that ENIG performs better than ImAg for the package side surface finish. In all cases, SAC305 solder alloy using ImAg, ENIG, or ENEPIG has a longer lifetime than SAC105, under both with/without thermal aging. The continuous growth of Cu–Sn IMC layer (SAC/ImAg systems) and Cu–Ni–Sn IMC layer (SAC/ENIG/ENEPIG systems) on solder joints finally result in fatigue failures. Under the help of embedded Ag_3_Sn particles which form in the solder during aging, higher Ag content solder balls (SAC305) perform better than SAC105. For 10 mm package size SAC105 BGA finished with ENIG and ENEPIG, cracks most likely start from the corner of the package side and then proceeds along the interface IMC and often into the solder bulk. Three factors are considered: package side IMC boundaries, Ag_3_Sn particles, and grain boundaries.

## Figures and Tables

**Table 1:** 10 mm BGA Package.

BGA Package	
Package Size	10 mm × 10 mm
Die Size	5.0 mm × 5.0 mm
Ball/Lead Count	360
Pitch	0.4 mm
Ball Allignment	Perimeter
Pad Finish	ENIG, ENEPIG, ImAg

**Table:** (**a**)

Solder Type	SAC 105		
Aging Period	0	125 °C/6 M	125 °C/12 M
ImAg	8	28	15
ENIG	5	15	10
ENEPIG	5	15	10

**Table:** (**b**)

Solder Type	SAC 305		
Aging Period	0	125 °C/6 M	125 °C/12 M
ImAg	8	28	15
ENIG	5	15	10
ENEPIG	5	15	10

**Table:** (**a**)

Solder Type	SAC 105					
Aging Condition	0		125 °C/6 M		125 °C/12 M	
Weibull Parameter	η	β	η	β	η	Β
ImAg	2419	3.515	1116	3.469	1079	4.079
ENIG	3422	2.683	2284	5.733	2022	7.059
ENEPIG	3536	3.503	2179	5.143	2061	4.308

**Table:** (**b**)

Solder Type	SAC 305					
Aging Condition	0		125 °C/6 M		125 °C/12 M	
Weibull Parameter	η	β	η	β	η	Β
ImAg	3329	4.364	1740	3.456	1551	3.729
ENIG	3974	2.072	2894	1.986	2671	2.395
ENEPIG	4661	3.831	2635	4.202	2536	2.651
